# Endorsement

**Published:** 1881-05

**Authors:** 


### ENDORSEMENT.

We published in the April number of The Homœopathic Physician, a letter from Dr. Pomeroy in which it was urged that homœopaths should endorse the eclectics by attending their society meetings, by writing for their journals, etc. The argument was that only the ignorant need teaching, only the sick need the physician, etc. This is true, but the teacher avails not unless the pupil studies, nor does the physician cure those who will not heed his advice. To continue the argument we would ask, does the teacher award certificates, endorsing their ability and knowledge, to scholars who have *not* those qualities? No! Why, then, should the homœopath endorse the eclectic, and so declare to the world that he is an able and qualified homœopathic physician?

For these and other reasons we feel constrained to place on record our disapproval of Dr. Pomeroy's conclusions. Dr. Pomeroy says the American Institute of Homœopathy needs instruction and endorsement. Now, though we do not deny for an instant that the Institute needs instruction, we do most emphatically deny that it will profit by such attendance and instruction. The Institute was founded by pure homœopaths and all the instruction there given was *at first* pure and able; *yet* it has degenerated into eclecticism! Would it have done so if teaching could have benefitted it?

Again, every attempt to instruct said Institute in pure homœopathy has been met in the past by ridicule and abuse. At the meeting of this Institute of Homœopathy (?) held June, 1879, Dr. McManus (Baltimore) related his experience with pure homœopathy. What was the result? Was any one impressed with his excellent success? Was increased study of the "Organon"

of Samuel Hahnemann the result? The only apparent answer was this, from a member: "I do not believe you ever cured a case of any kind of disease with the 30th attenuation of medicine"!*

At a later time, Dr. Berridge read a paper inculcating the necessity of continued study of the "Organon," but it met with extreme abuse and ridicule. The members were very indignant that it should be said they were not familiar with the "Organon." Thus, by their continued rejection of pure teaching, showing they do *not want to be taught, they feel no need of instruction!* In fact, there is scarcely one of that eclectic company who does not believe that he could write a much better book than the "Organon"! Can such men be taught? "Seest thou a man wise in his own conceit? there is more hope of a fool than of him."—(Proverbs, xxvi, 12).

Have you ever, in all your long experience, known a man—eclectic or allopath—to be converted to homœopathy in any other way than by studying the "Organon" and following its precepts? Attempt to teach this *noble* body of *homœopaths* and you meet the fate of Drs. McManus and Berridge.

But one can write sound papers for their journals, you will say. Yes, you can, and to the waste basket they go! A noted homœopath furnished a so-called homœopathic journal with an article showing the success of homœopathic treatment in a grave disease. This paper was *refused* and ridiculed as "Munchausenism"!

You moreover believe that eclectics should be "endorsed." Let us put the case plainly. Suppose there are in your city six eclectics; you are well known there as a successful and true homœopath. You meet these six eclectics in society meetings, in consultations, etc., etc. Well, a patient of yours is suddenly taken ill, your office is far off, so Dr. A., whom you have "*endorsed*," is called in; he gives *Quinia*, *Morphia*, etc., patient dies! Have you done right in giving such *homœopathic* (!)

* See "Transactions of Institute," 1879, p. 272.

treatment to your clients? Again, suppose you *endorse* these eclectics; a patient, worn out with allopathic treatment, determines to try, for the first time, homœopathy. They say, "We have heard of this and that case cured by Dr. P., but he is too far off, and as we know Dr. A. belongs to the same society, and affiliates with Dr. P., he must practice as does Dr. P., hence we will try him." They do so. Dr. A. applies his eclectic methods, the patient is either disgusted with *such homœopathy* and returns to the allopathic fold or worse, dies from it. Are you willing to *endorse* such every day occurrences? Does not homœopathy suffer sadly from such "*endorsement*"? Are not innocent laymen imposed upon by such "*endorsement*"?

"Be ye not unequally yoked together with unbelievers: for what fellowship hath righteousness with unrighteousness? and what communion hath light with darkness? . . . . or what part hath he that believeth with an infidel?"—(II. Corinthians, vi, 14, 15).

